# Parvovirus B19-induced vascular damage in the heart is associated with elevated circulating endothelial microparticles

**DOI:** 10.1371/journal.pone.0176311

**Published:** 2017-05-22

**Authors:** Katrin Bachelier, Susanne Biehl, Viktoria Schwarz, Ingrid Kindermann, Reinhard Kandolf, Martina Sauter, Christian Ukena, Ali Yilmaz, Karen Sliwa, Claus-Thomas Bock, Karin Klingel, Michael Böhm

**Affiliations:** 1 Klinik für Innere Medizin III, Kardiologie, Angiologie und Internistische Intensivmedizin, Universitätsklinikum des Saarlandes, Homburg/ Saar, Universität des Saarlandes, Saarlandes, Germany; 2 Universitätsklinikum Tübingen, Abteilung Molekulare Pathologie, Institut für Pathologie und Neuropathologie, Tübingen, Germany; 3 Universitätsklinikum Münster, Department für Kardiologie und Angiologie, Münster, Germany; 4 Hatter Institute for Cardiovascular Research in Africa and MRC Inter-Cape Heart Group, Faculty of Health Sciences, University of Cape Town, Cape Town, South Africa; Klinikum Region Hannover GmbH, GERMANY

## Abstract

**Background:**

Diagnosis of viral myocarditis is difficult by clinical criteria but facilitated by detection of inflammation and viral genomes in endomyocardial biopsies. Parvovirus B19 (B19V) targets endothelial cells where viral nucleic acid is exclusively detected in the heart. Microparticles (MPs) are released after cell damage or activation of specific cells. We aimed to investigate whether circulating endothelial MPs (EMPs) in human and experimental models of myocarditis are associated with B19V myocarditis.

**Methods:**

MPs were investigated in patients with myocarditis (n = 54), divided into two groups: B19V+ (n = 23) and B19V- (n = 31) and compared with healthy controls (HCTR, n = 25). MPs were also investigated in B19V transgenic mice (B19V-NS1+) and mice infected with coxsackievirus B3 (CVB3). MPs were analyzed with fluorescent activated cell sorting (FACS).

**Results:**

In human samples, EMP subpopulation patterns were significantly different in B19V+ compared to B19V- and HCTR (p<0.001), with an increase of apoptotic but not activated EMPs. Other MPs such as platelet- (PMPs) leukocyte-(LMPs) and monocyte-derived MPs (MMPs) showed less specific patterns. Significantly different levels of EMPs were observed in transgenic B19V-NS1+ mice compared with CVB3-infected mice (p<0.001).

**Conclusion:**

EMP subpopulations are different in B19V+ myocarditis in humans and transgenic B19V mice reflecting vascular damage. EMP profiles might permit differentiation between endothelial-cell-mediated diseases like myocardial B19V infection and other causes of myocarditis.

## Introduction

Myocarditis is a non-ischemic inflammatory heart disease, which is potentially leading to severe heart failure and death [1,2]. Clinical manifestations vary with a broad spectrum from mild symptoms to cardiogenic shock [1–4], sometimes with the need for heart transplantation [5]. Myocarditis can result from common viral infections and post-viral immune-mediated responses [6]. Parvovirus B19 (B19V), a non-enveloped single-stranded DNA virus, belongs to the genus of erythroviruses, invades and replicates in erythroid precursor cells and endothelial cells [7]. Since diagnosis can be difficult, endomyocardial biopsy (EMB) with immunohistology is needed to define inflammation and molecular patterns in order to characterize the types of viral infection [8,9]. Many studies have detected B19V genomes in EMB from patients with acute and chronic myocarditis [10] with diastolic dysfunction [11] and peripartal [6] cardiomyopathy. The high prevalence of B19V (30–35%) in dilated cardiomyopathy (DCM) suggests that DCM could develop from previous B19V-associated myocarditis [11,12]. However, many individuals (80%) at the age of 60 carry B19V- that its specifity has been questioned [13]. It has been shown that cardiac endothelial cells (ECs) but not myocytes are the B19V-specific targets providing expression of the blood-group P-antigen serving as a cellular receptor for B19V [14] allowing persistence of B19V in ECs leading to endothelial cell apoptosis [15] EMPs are released from cellular membranes during cell activation and apoptosis [16] and predict flow-mediated dilatation, cardiovascular events in rheumatoid arthritis [17] with endothelial dysfunction, predicts outcomes in acute coronary syndromes [18] and allow differentiating peripartal cardiomyopathy from normal pregnancy and other causes of heart failure [19]. It is unknown whether EMPs can differentiate among inflammatory cardiac diseases. We investigated circulating EMPs in patients with B19V+ and B19V- myocarditis to explore whether endothelial and myocardial damage can be distinguished. We compared the human findings with mouse models of transgenic B19V-NS1 mice or CVB3 myocarditis and controls.

## Methods

### Study design

#### Patients

Blood samples were obtained from patients with clinical evidence for myocarditis (n = 54), divided into two groups after endomyocardial biopsy (EMB), B19V+ (n = 23, EF 53±18%) and B19V- (n = 31, EF 46±21%) and then compared with healthy controls (HCTR, n = 25). All patients underwent left ventricular EMB and histological, immunohistological and molecular workup as previously described [5,9,19]. After informed consent, 10 ml peripheral venous blood was sampled from each of the 79 enrolled subjects. Demographic and clinical data are summarized in Table 1. Controls were age-matched volunteers who had no cardiovascular disease. They had been recruited during 2008–2009 for several studies. The study was approved by the appropriate ethics committee (Ethikkommission der Universität des Saarlandes, Nr. 122/09). All patients gave written informed consent to include their data in the study.

**Table 1:** Clinical parameters of human samples.

	B19V+ (n = 23)	B19V+ (p-value vs HCTR)	B19V- (n = 31)	B19V- (p-value vs HCTR)	HCTR (n = 25)
Clinical Parameters					
Mean Age [yrs] ± SD	55 ± 13	*0*,*775*	60 ± 11	*0*,*505*	51 ± 6
Gender [m:w]	10:13	*-*	21:10	*-*	0:14
Virus infection (EMB)					
HHV6	0	*-*	3	*-*	-
EBV	0	*-*	1	*-*	-
B19V	23	*-*	0	*-*	-
No Virus	0	*-*	27	*-*	-
Echocardiographic parameters					
LVEDD [mm]	62 ± 16	*0*,*693*	64 ± 21	*0*,*690*	53 ± 16
LVESD [mm]	50 ± 14	*0*,*409*	51 ± 15	*0*,*456*	38 ± 5
IVSD [mm]	14 ± 6	*0*,*610*	13 ± 2	*0*,*409*	11 ± 1
IVDD [mm]	11 ± 4	*0*,*820*	14 ± 3	*0*,*297*	10 ± 2
LVPWD [mm]	10 ± 4	*0*,*820*	11 ± 3	*1*,*000*	11 ± 2
LVPS [mm]	15 ± 3	*0*,*245*	16 ± 2	*0*,*103*	10 ± 3
LVEF [mm]	53 ± 18	*0*,*313*	46 ± 21	*0*,*251*	79 ± 18
Laboratory parameters					
CK [U/I]	155 ± 80	*0*,*493*	132 ± 67	*0*,*674*	99 ± 25
CK-M [U/I]	45 ± 12	*0*,*010*	41 ± 9	*0*,*010*	<14 ± 0
ASAT [U/I]	85 ± 18	*0*,*006*	55 ± 12	*0*,*117*	32 ± 6
ALAT [U/I]	90 ± 13	*0*,*002*	48 ± 8	*0*,*388*	37 ± 10
LDH [U/I]	301 ± 267	*0*,*601*	315 ± 210	*0*,*527*	160 ± 75
Creatinine [mg/dL]	1.12 ± 0.81	*0*,*789*	1.21 ± 0.75	*0*,*881*	1.34 ± 0.24
Troponin [ng/mL]	1.6 ± 1.0	*0*,*104*	1.2 ± 0.4	*0*,*010*	<0.01
NT-Pro-BNP [pg/mL]	1498 ± 2526	*-*	2285 ± 1847	*-*	0 ± 0
CRP [mg/mL]	46 ± 72	*-*	50 ± 11	*-*	0 ± 0

Basic characteristics of patients with DCM with positive EMB for B19V (B19V+), negative EMB for B19V (B19V-) and healthy controls (HCTR). LVEDD = left ventricular enddiastolic diameter, LVESD = left ventricular endsystolic diameter, IVSD = interventricular endsystolic diameter, IVDD = interventricular enddiastolic diameter, LVPWD = left ventricular posterior wall diameter, LVPS = left ventricular posterior septal diameter, FS = fractional shortening, LVEF = left ventricular ejection fraction, CK = creatine kinase, CK-M = creatine kinase muscle, ASAT = aspartate transaminase, ALAT = alanine transaminase, LDH = lactate dehydrgenase, NT-Pro-BNP = N-terminal pro brain natriuretic peptide, CRP = c-reactive protein, n.d. = not determined.

Plus-minus values are means ± Standard Deviation (SD); LVEDD: Left ventricular enddiastolic diameter; LVESD: Left ventricular endsystolic diameter; IVSD: Interventricular endsystolic diameter; IVDD: Interventricular enddiastolic diameter; LVPWD: Left ventricular posterior wall diameter; LVPS: Left ventricular posterior septal diameter; LVEF: Left ventricular ejection fraction; CK: Creatinine kinase; CK-M: Creatinine kinase muscle; ASAT: Aspartate transaminase; ALAT: Alanine transaminase; LDH: Lactate dehydrogenase; NT-Pro-BNP: N-terminal pro brain natriuretic peptide; CRP: C-reactive protein; HHV-6 human herpes virus type 6, EBV: Epstein-Barr virus, B19V: parvovirus B19

#### Generation of conditional transgenic B19V-NS1 mouse lines

B19V DNA was isolated from deparaffinized myocardial tissues of patients with fatal B19V-associated myocarditis as described previously to generate transgenic mice (accession no. AY768535 and AF162273) [20]. C57BL/6 mice were used for the generation of transgenic B19V mice (B19V-NS1) as well as control mice. For details refer to S1 Appendix.

#### Murine CVB3 myocarditis

CVB3 used in this study was derived from the infectious cDNA copy of the cardiotropic Nancy strain, and virus stocks were prepared as previously described [21]. C57BL/6 mice were infected with CVB3. Details can be found in S1 Appendix.

#### Isolation of microparticles

Microparticles were isolated as described previously [19]. Details can be found in S1 Appendix.

#### Flow cytometry

The details of the technique are described elsewhere [19] and summarized in the S1 Appendix. EMPs and PMPs both express CD31. For exact delineation of CD31-positive EMPs and not platelet-derived CD31-positive MPs, CD42b-negative MPs were analyzed in platelet-free plasma.

### Statistical analysis

Data are expressed as mean ± standard error of the mean (SEM). Continuous variables were tested for normal distribution with the Kolmogorov-Smirnov test and compared using a two-way ANOVA test, followed by a two-sided Bonferroni post-hoc testing. Tests for equal variance normality were performed using the Levene Median test. A p-value of <0.05 was considered statistically significant. Assumptions of normality and equal variance were automatically tested using the statistic program. Normal distribution of the parameters (NT-proBNP, CRP) was tested here using a Kolmogorov-Smirnov test. Both parameters showed normal distribution and are reported as mean +/- SD. Statistical analyses were performed using SigmaStat version 3.5. All data analyses and event classifications were performed by investigators blinded to the microparticle-status of patients and controls.

## Results

The clinical and demographic data of the studied individuals are given in Table 1. FACS-analysis could be performed in all samples with myocarditis and were compared with healthy controls (n = 25). Controls had normal left ventricular fractional shortening and diameters without any differences between the groups. No significant differences occurred between B19 virus positive (B19V+) or negative (B19V-) hearts.

### Human microparticles

#### Endothelial MPs (EMPs)

**Fig 1:**
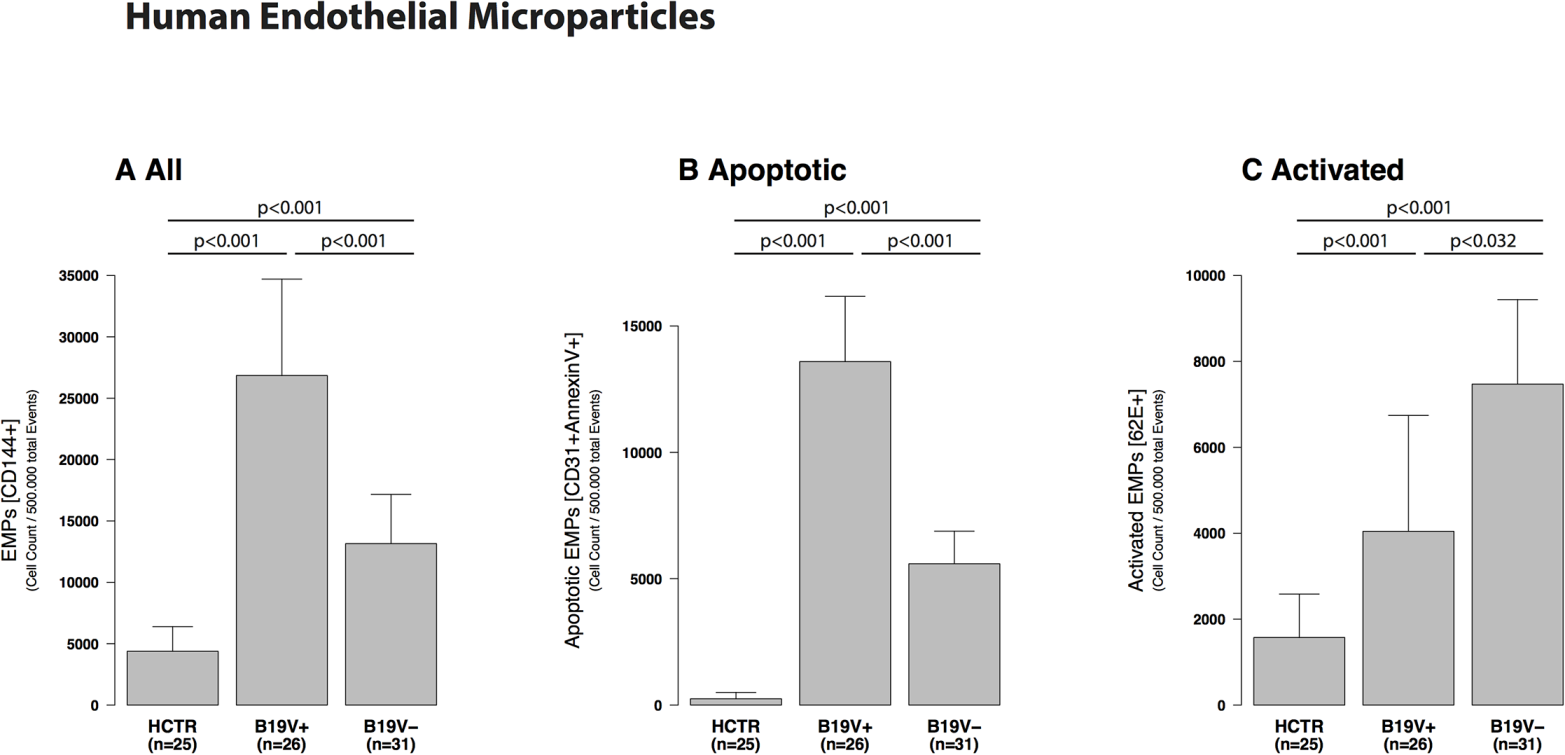
Human endothelial microparticles. Human endothelial microparticles (EMPs) from patients with myocarditis divided into B19V+ and B19V- patients compared with age-matched healthy controls (HCTR). A: EMPs were significantly increased in B19V+ patient samples compared to B19V- and HCTR. B19V- had less increased EMP, only significant versus HCTR. B: CD31/AV-positive EMPs represent apoptotic EMPs. Apoptotic EMPS were significantly higher detectable than activated EMPs in B19V+ than in all other groups (p<0.001). C: CD62E-positive EMPs represent activated EMPs. B19V - samples had lower levels but significantly elevated activated EMPs compared to HCTR (p<0.001).

Subpopulations of EMPs (CD144+) were significantly different in B19V+ compared to B19V- and HCTR (p<0.001, Fig 1A). The increase in B19V+ was due to an increase of apoptotic (Fig 1B, CD31+AV+) but not activated EMPs (Fig 1C, CD62E+) reflected in a lower CD62E/CD31 ratio (Table 2).

**Table 2:** CD62 / CD31 ratio in endothelial microparticles.

	CD62E / CD31 Ratio SEM	p-value vs. control	p-value vs. max. of apoptosis
Humans			(vs. B19V+)
B19V+	0.4 ± 0.8	<0.001	-
B19V-	1.4 ± 0.2	1.000	<0.001
HCTR	1.4 ± 0.1	-	<0.001
Mice			(vs. B19V+ 2wks)
C57 / BL6	1.4 ± 0.9	-	0.040
B19V no Doxy	1.4 ± 1.1	1.000	0.066
B19V+ 2wks	0.3 ± 0.7	0.040	-
B19V+ 4wks	0.8 ± 0.2	0.115	0.123
B19V+ 6wks	0.9 ± 0.2	0.240	0.071

CD62E/CD31-ratio is used as an index of activation (high ratio, ≥4) or apoptosis (low ratio, <0.4) for distinguishing between apoptotic or activated EMP generation. The increase of EMPs in our study, either in humans or mice, was due to significantly elevated apoptotic EMPs (CD31^+^) and not activated EMPs (CD62E^+^) reflected by the lower CD62E^+^/CD31-ratio. In CBV3+ samples the highest maximum of apoptosis was shown to be after 2 days post infectionem wheras it was reached in B19V+ 2 weeks after induction. Comparing the maximums of both groups transgenic B19V-NS1-mice with induction by doxycycline demonstrated a significant ratio (p = 0.004) indicating a higher endothelial apoptosis.MP = microparticles, EMPs = Endothelial microparticles, PMP = Platelet-derived microparticles, MMPs = Monocyte microparticles, LMPs = Leucocyte microparticles), m = mean, SD = standard error.

#### Platelet-derived MPs (PMPs)

PMPs (CD62P+CD42b+AnnexinV+) increased in B19V+ compared to B19V- patients and healthy controls (HCTR, p<0.001, S1A Fig). PMPs in B19V-negative patients were similar in healthy controls. Apoptotic PMPs were significantly increased in B19V+ compared to B19V- (p<0.001) and to healthy controls (p<0.024) (S1B Fig). Activated PMPs were slightly elevated in B19V- compared with B19V+ (p = 0.004) and HCTR (p = 0.001), as demonstrated in S1C Fig. In B19V+, activated PMPs were slightly altered in comparison with HCTR (p = 0.023).

#### Monocyte MPs (MMPs) and leukocyte MPs (LMPs)

MMPs (CD14+AnnexinV+) were increased in both, B19V+ and B19V- in contrast to healthy controls (p<0.001 and p<0.003, S2A Fig) without significance between themselves. LMPs (CD45+AnnexinV+) were significantly increased in B19V+ compared to B19V- (p<0.011) and healthy controls (p<0.004) as seen in S2B Fig.

### Microparticles in mice

#### Endothelial MPs (EMPs)

**Fig 2:**
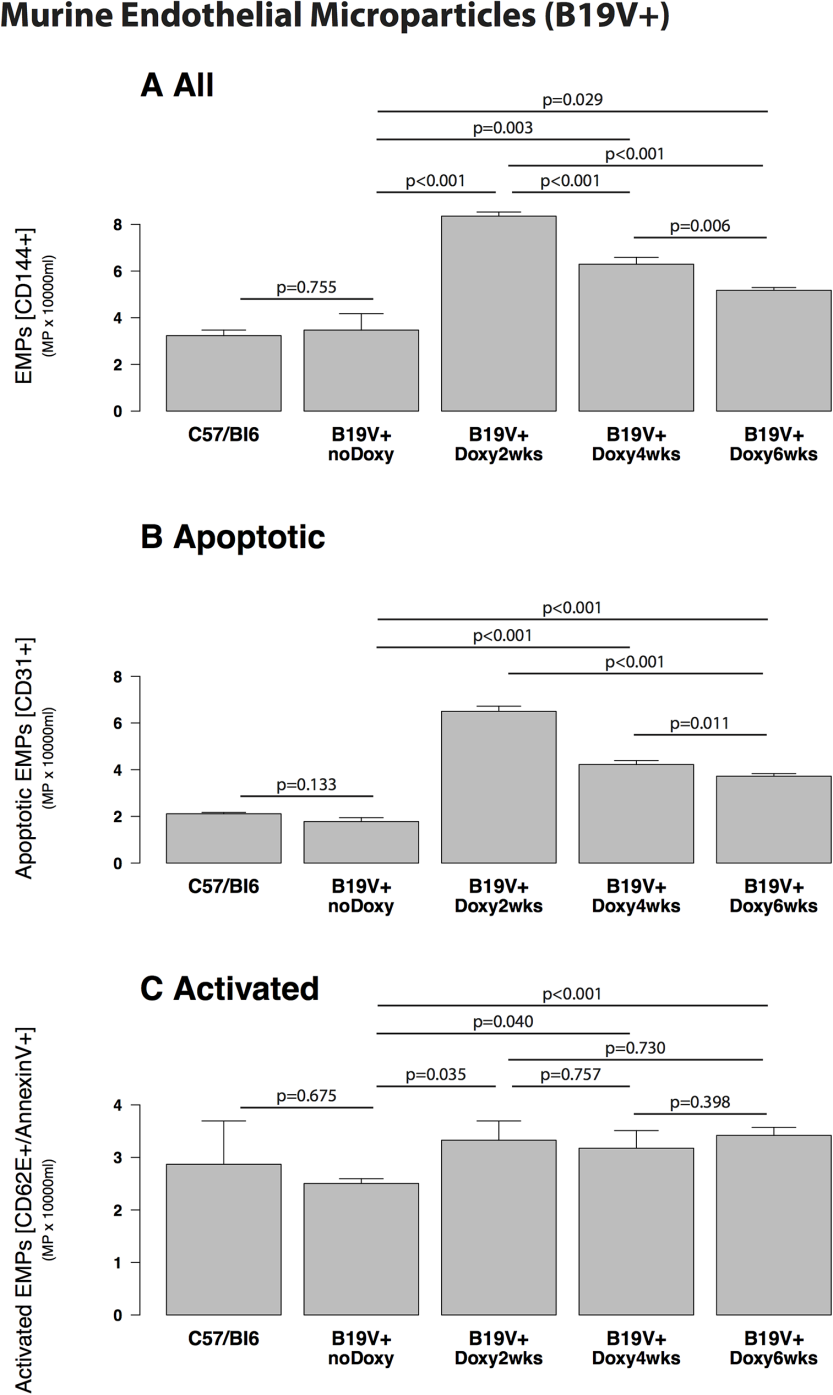
Murine endothelial microparticles (B19V- transgenic mice). Murine endothelial microparticles (EMPs) in transgenic B19V-NS1-mice with induction by doxycycline (B19V+) after 2, 4 and 6 weeks p.i. compared with controls (C57/Bl6 and transgenic B19V-NS1 mice without doxycyclin). A: EMPs in C57/Bl6 mice compared to transgenic B19V-NS1-mice without doxycyclin showed about the same EMP numbers (p = 0.775). EMPs were significantly increased in transgenic B19V-NS1-mice with doxycyclin after 2, 4 and 6 weeks compared to controls such as C57/Bl6 (p<0.001) and transgenic B19V-NS1-mice without doxycylin (p<0.001, p = 0.003 and p = 0.029). The increase had its maximum after two weeks with a decline after four weeks. B: The increase of EMPs was due to apoptotic EMPs. C: Activated EMPs were not different between the groups.

**Fig 3:**
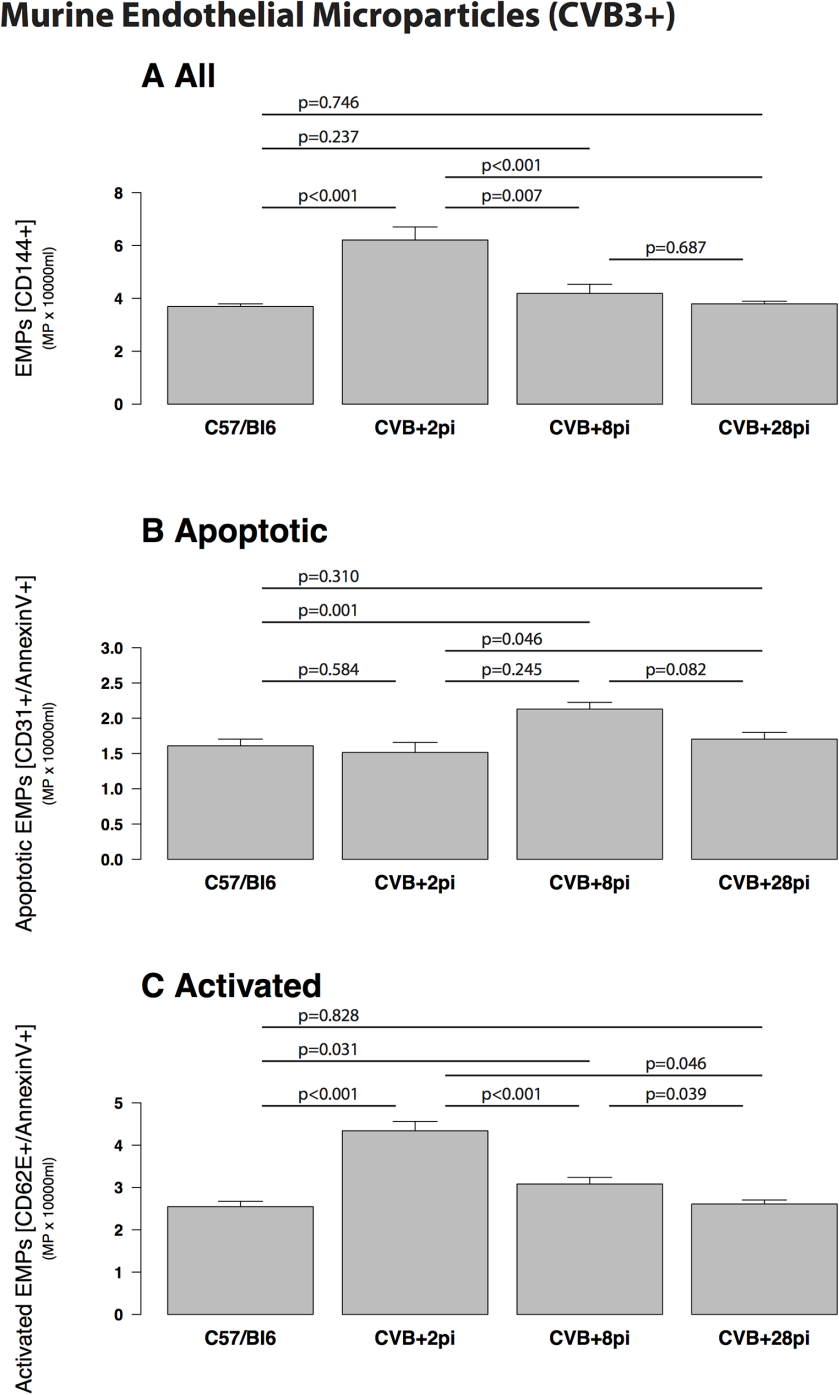
Murine endothelial microparticles (CVB3+ infected mice). Murine endothelial microparticles (EMPs) in CVB3+ infected mice (CVB3+) after 2, 8 and 28 days p.i. compared with controls (C57/Bl6). A: EMPs were increased in CVB3+ mice two days p.i. (p<0.001 vs. control) with a decline in the following 6 days (p<0.001 vs. control) and 28 days p.i. (p<0.001 vs. control). B: The increase of EMPs was due to apoptotic EMPs. C: Activated EMPs were not different between the groups.

EMPs measured in C57BL/6 control mice compared to conditional transgenic B19V-NS1-mice without induction by doxycyclin as negative control were not changed (p = 0.755, Fig 2A). EMPs significantly increased in transgenic B19V-NS1 mice 2, 4 and 6 weeks after induction with doxycycline compared to controls (C57BL/6, p<0.001, Fig 2A) and B19V-NS1-mice without doxycylin (p<0.001, p = 0.003 and p = 0.029, Fig 2A). The increase had its maximum after two weeks (8.3 ± 0.14 x103/ml) with a decline after four weeks (Fig 2A). Changing of EMPs was due to apoptotic EMPs as shown in Fig 3B and not to activated EMPs (Fig 2C).

Mice infected with coxsackievirus B3 (CVB3) were studied for comparison. A small increase of EMPs was detectable in CVB3-infected mice at the early stage of infection (2 days p.i. (6.19 ± 0.5 x103/ml, p<0.001 vs. control) with a rapid decline during acute infection at 8 days p.i. later at 28 days p.i (Fig 3). Comparing both model systems, EMPs were significantly more increased in B19V-NS1-mice compared to CVB3-infected mice 2 days p.i. (p = 0.009, not shown).

#### Other MPs

PMPs measured in C57BL/6 control mice compared to transgenic B19V-NS1-mice without doxycyclin showed comparable levels (p = 0.463, not shown). PMP of B19V-NS1 after induction and of CVB3 mice after infection did not rise significantly (not shown). MMPs and LMPs measured in C57BL/6 control mice compared to transgenic B19V-NS1-mice without doxycyclin showed about the same levels (p = 0.768, not shown).

#### EMP apoptosis and activation

The ratio of CD62E/CD31 EMPs reflects endothelial activation versus apoptosis. The changed patterns of EMPs in humans (Fig 1) or mice (Fig 2) was due to significantly elevated apoptotic EMPs (CD31+) and not activated EMPs (CD62+). This is shown by the lower CD62/CD31 ratio (Table 2). Induced transgenic B19V-NS1-mice demonstrated a significantly elevated ratio (p = 0.004, Table 2) indicating a higher endothelial apoptosis compared to human B19+ hearts.

## Discussion

EMPs patterns were changed in humans with B19V myocarditis and in transgenic B19V-mice compared to healthy controls and control mice, respectively. In CVB3-infected mice, there were no or minor changes in EMPs, but increased levels of LMPs.

B19V DNA is the most frequent viral genome observed in endomyocardial biopsies (EMBs) with left ventricular dysfunction [2,11]. Previously, we found that myocardial endothelial cells but not myocytes are B19V-specific target cells [22]. B19V infects endothelial cells of small myocardial blood vessels resulting in impairment of myocardial endothelial dysfunction and impairing myocardial microcirculation [23,24]. Consistently, the presence of B19V-viral genome was associated with endothelial dysfunction and diastolic dysfunction [11] in patients with clinical signs of myocarditis. Patients can present with coronary vasospasm and atypical chest pain in patients with clinical signs of myocarditis and biopsy-proven myocarditis with virus persistence in the absence of significant coronary artery disease [25]. Herein, we found changed patterns of EMPs as a detectable marker reflecting endothelial damage, which were elevated in B19V+ patients but not in patients with B19V- myocarditis.

In order to add plausibility, we investigated transgenic B19V mice. Also, in this B19V associated model, an increase of EMPs was detected. It was accompanied by high levels of platelet-derived PMPs and LMPs, which could reflect the response to vascular damage reflected by significantly elevated EMPs. It is known that PMPs generated from apoptotic human platelets induce human monocyte chemotaxis and polarization into resident M2 monocytes, implying that these MPs possess immunomodulating properties [26]. MPs could act as signal transducers taking a critical role in mediating autoimmunity processes in the heart [27]. Activation or apoptosis of endothelial cells can lead to specific MP type formation, which can be differentiated by their specific marker expression patterns. The increased CD62E/CD31 ratio reported herein, suggests that apoptosis is an important mechanism for EMP release in B19V-induced heart disease.

In comparison to the murine model of B19V-induced heart disease, CVB3-infected mice showed higher levels of LMPs. CVB3 is known to infect primarily cardiomyocytes, and due to extensive virus replication, a rapid cytolysis of these cells occurs. Before reaching their cardiac target cells, the cardiomyocytes, CVB3 transmigrates through the endothelium for a short period of time during viremia [28]. The rise of EMPs in CVB3-infected mice observed in our study is likely reflecting diapedesis with penetration of virus through endothelial layers and affection of endothelial cells leading to a temporary vascular damage [28]. Subsequent antiviral immune responses might be an explanation for the activation of other MP types in these animals. Our measurements of MPs in CVB3-infected mice revealed a significant increase of inflammatory MPs such as MMPs and LMPs during acute myocarditis (8 days p.i) staying at higher levels at later stages of the disease (28 days pi). MCP-1 is known to mediate migration of monocytes into virus-affected sites [29]. Elevation of MCP-1 levels at the initial presentation in patients with acute myocarditis was significantly correlated with the severity and prognosis [30]. The high levels of MMPs in our study are in agreement with the known activation of monocytes in the inflammatory response.

One limitation of the present study is the absence of coxsackievirus B infection in humans in this study. Unfortunately, human samples with CVB-myocarditis are rare. Therefore, the murine model of CVB3 myocarditis was used as it reflects human enteroviral myocarditis with regard to myocardial damage and virus-induced immune response [31]. Endomyocardial biopsies are usually taken in these patients at different time points after onset of myocarditis. Therefore, we cannot reconstruct the timing of the phases of inflammatory myocardial disease in our patients. The severity and outcome of the disease in different mouse models as well as the relative contributions of direct viral and inflammation-mediated mechanisms to the pathogenesis of the disease show apparently the same high variability as seen in humans [30]. Finally, we cannot exactly define whether the changed EMP patterns are derived from, the heart or also from the peripheral circulation. Since B19V infection is a systemic disease, apoptotic EMPs could also be derived from other compartments than the heart.

Our data provide first evidence that differential endothelial microparticle changes are detected in different virus-associated heart diseases. Differences in the subtypes of MPs can be attributed to specific myocardial virus infections targeting different cell types. B19V persists in the endothelium [15] and, thus, induces endothelial damage, while CVB3 targets myocytes and shows less pronounced transient endothelial reactions [25,28]. Both viral infections can finally lead in chronic myocardial disease and myocardial fibrosis with end-stage heart failure [6]. However, independently from the responsible virus leading to myocarditis, apoptotic processes are involved as indicated via AnnexinV positive MPs, which are detectable in CVB3- and B19V- induced myocarditis. Taken together, these data strengthen the notion that apoptosis may play a pivotal role in acute and chronic myocarditis. There is an increased awareness of the importance of myocarditis being a meaningful cause for DCM and heart failure. Substantial progress in diagnosis and management has been made over the past decade. However, myocarditis remains a diagnosis of exclusion and diagnosis is often delayed with consecutive late initiation of arising therapies like immunosuppressive or antiviral treatment [32]. In this context and in the light of unreliable serodiagnostic [33], there is a clinical need for diagnostic biomarkers. Microparticle profiling could potentially become a valuable tool facilitating earlier diagnosis. EMPs are derived from extracellular vesicles, which are not only presenting debris from cellular damage, but are carrying proteincytokines, MRAs and non-coding MRAs to other target cells and, thus, are presenting one mechanism of intracellular communication. Future researches will have to address not only their patterns and different etiologies of inflammatory myocardial disease, but their function which could relate a progression of myocardial disease, but also protective mechanisms [34,35].

It is concluded that microparticle profiles vary between different myocardial diseases and could facilitate early differential diagnosis between endothelial-cell-mediated disease due to B19V and other causes of myocarditis and pave the way to early diagnoses and potentially to early initiation of treatment.

## Supporting information

S1 Appendix Methods. (DOCX)

S1 Fig Human platelet-derived microparticles (PMPs). Human platelet-derived microparticles (PMPs) in patients with myocarditis divided into B19+ and B19V- patients and compared with age-matched healthy controls (HCTR). The B19V- group consisted of either no virus detection or HHV6+ and EBV+ samples. A: PMPs were significantly increased in B19V + patient samples compared to B19V- and HCTR. B19V- had increased EMP levels as well, but not significant versus HCTR. B: CD42b-AV+ PMPs represent apoptotic PMPs. Apoptotic PMPs were significantly higher detectable than activated PMPs in B19V+ and B19V-. C: CD62P+ PMPs represent activated PMPs.
(TIFF)

S2 Fig Circulating inflammatory MPs. Monocyte-derived microparticles (MMPs,A) and leukocyte-derived microparticles (LMPs,B) in patients with myocarditis divided into B19V+ and B19V- patients and then compared with age-matched healthy controls (HCTR). The B19V- group consisted of either no virus detection or HHV6+ and EBV+ samples. A: MMPs were increased in both, B19V+ and B19V - in contrast to healthy controls (p<0.001 and p<0.003) but no significance between themselves. B: LMPs were significantly increased in B19V+ compared to B19V- (p<0.011) and healthy controls (p<0.004).
(TIFF)
