# Supplementary material for: Parvovirus B19-induced vascular damage in the heart is associated with elevated circulating endothelial microparticles
Source: PLoS One. 2017 May 22;12(5):e0176311. doi: 10.1371/journal.pone.0176311 (PMC5439674; doi:10.1371/journal.pone.0176311)
Supplement: S1 Appendix — (DOCX) [file pone.0176311.s001.docx]

**Supporting information**

**S1 Appendix. Methods**

**Generation of conditional transgenic B19V-NS1 mouse lines**

The NS1 region of the B19V genome was amplified by PCR using high-fidelity polymerase (Pwo Super Yield DNA polymerase; Hofmann-LaRoche, Basel, Switzerland). To achieve controlled expression of the B19V NS1, the NS1 gene was cloned under the control of the tetracycline-controlled transcriptional activator rtTA-VP16 [19]. The resulting NS1-specific amplicons were subcloned into the mammalian expression vector pWHE146 under the control of the endothelium specific ICAM2 promoter and bidirectional promoter-response-element to rtTA-VP16 system (kindly provided by Prof. Dr. Hillen, University of Erlangen, Germany). The bidirectional promoter is capable for the expression of the viral gene and of the reporter gene eGFP. The newly generated constructs were sequenced and the B19V-NS1 and reporter gene expression was checked in cell culture experiments.

C57BL/6 mice which were obtained from Charles River (Willmington, USA), were used for the generation of transgenic B19V mice. Mice were bred and maintained according to established guidelines and in accordance with the German animal protection law and with permission of the Regierungspräsidium Tübingen (PA1/11). pWHE146-B19V-NS1 and pWHE163 constructs were injected into the pronucleus of fertile oocytes and the embryos transferred into mock pregnant animals. After genotyping of the founder animals, the pWHE146-B19V-NS1 and the pWHE163 mice were bred. Both animal lines were hybridized to get the double transgenic animals carrying the B19V-NS1 gene under control of the tetracyclin-dependent rtTA response promoter in endothelial cells. Mice were induced with the tetracyclin-derivate doxycyclin 1mg/ml in drinking water containing 2% of sugar ad libitum. After 2, 4, 6 weeks mice were sacrificed and blood was taken for further analysis.

**Murine CVB3 myocarditis**

C57BL/6 mice were kept under specific pathogen-free conditions at the animal facilities of the Department of Molecular Pathology, University Hospital Tübingen, and experiments were conducted according to the German animal protection law and with permission of the Regierungspräsidium Tübingen (PA 2/10). Four- to 5-week-old mice were infected intraperitoneally with 1x10^5^ plaque-forming units of purified CVB3 as described [20]. At different time points after infection (2, 8, 28 days p.i.), mice were sacrificed, and hearts, spleens, and serum were collected for analysis. Uninfected C57BL/6 mice (n=6) served as controls.

**Isolation of microparticles**

Isolation of the MPs was performed using one unique protocol in our laboratory only in order to rule out variations in pre-analytic procedures. Whole blood in EDTA of all samples was centrifuged at 1.500g for 15 minutes to prepare platelet-rich plasma, centrifuged again for 2 minutes at 13.000g to obtain platelet-poor plasma within 30 minutes of blood sampling, and stored at -20°C for one week and afterwards at -80°C until analysis. Before storage aliquots of 125µl platelet-free plasma and 25µl platelet-enriched plasma sample sizes were assembled. Analysis was always performed after 28-35 days to minimize differences due to different long period of thawing time [18]. Total 125µl of platelet-poor plasma was aliquoted into five tubes (25µl each). On with fluorescent monoclonal antibodies (2µl each): phycoerythrin (PE)-labelled anti-CD31 (BD Biosciences, San Jose, CA), fluorescein isothiocyanate-labelled (FITC) anti-CD144 (R&D, Minneapolis), FITC-labelled anti-CD62E (BD Biosciences), FITC-labelled anti-CD14 (BD Biosciences), PE-labelled anti-CD45 (BD Biosciences) and allophycocyanin-labelled AnnexinV (BD Biosciences). PMPs were analyzed in platelet-rich plasma by incubation with FITC-labelled anti-CD62P, and allophycocyanin-labelled AnnexinV (all BD Biosciences). The samples were incubated at room temperature for 30 minutes with gentle shaking (orbital shaker, 120rpm). Phosphate-buffered saline buffer (0.20µm filtered for reducing background noise) was added to make the total volume 1ml, and the samples were then analyzed on flow cytometer (FACS Calibur, BD Biosciences) and Cell Quest Pro software to detect fluorescence, forward and sideward scatter. For exact delineation of CD31-positive EMPs and not platelet-derived CD31-positive MPs, CD42b-negative MPs were analyzed in platelet-free plasma. For definition of pure EMPs, CD144 was chosen additionally, as this marker is selectively expressed on endothelial cells [21]. For distinguishing between activation and apoptosis in terms of EMP generation, CD62E and CD31 have been chosen. It is known that EMPs expressing constitutive markers, such as CD31, are markedly increased in apoptosis, whereas those expressing inducible markers, such as CD62E, are increased in activation. Therefore, phenotypic assessment of EMPs could provide relevant information reflecting the nature of endothelial injury. Stability of EMPs has been reported before [18]. MMPs were analysed by incubation with FITC-labelled anti-CD14, LPM1 with PE-labelled anti-CD45.

**Flow cytometry**

Flow cytometry was performed as described previously [18]. Microbeads from a FACS size-calibration kit (LB-30, Sigma, Munich, Germany) were used for size calibration. Logarithmic scale was implemented for forward scatter signal, side scatter signal and each fluorescent channel. Non-stained samples and isotype controls were used to discriminate true events from background noise, and to increase the specificity for MP detection. An isotype control antibody was used as a negative control in all measurements and subtracted from MP counts. In all our measurements, non-specific binding was accounted for less than 7% of total MPs. EMPs smaller than 1µm were quantified in subpopulations, that is, CD31+CD144+AV+, CD31+CD144+AV-, CD31+AV+, CD31+CD42b-AV+, and CD62E+ MPs. Both CD31 and CD144 are considered endothelial markers but CD144 is known to be a more endothelial-specific marker. Expression of constitutive markers such as CD31 is markedly increased in apoptosis, whereas those expressing inducible markers, such as CD62E, are increased in activation. For distinguishing between apoptotic and activated EMPs the ratio CD62E/CD31 was calculated [30]. PMPs smaller than 1µm were quantified in a specific population of CD42b+CD62P+AV+ microparticles. Values were reported as counts in 1µl platelet-poor or platelet-rich plasma (counts/500.000 events). Microparticles and calibrator beads (CAL) (10µm diameter) were visualized in a forward light scatter (FSC) and side-angle scatter (SSC). Microparticles were defined as events (size 0.1 to 1µm, R1) and then plotted in the “R2” window (upper left) on a FL/FSC fluorescence dot plot to determinate positively labelled MPs by specific antibodies. MP concentration was assessed by comparison to flowcount calibrator beads. Events beyond the gated MPs representing background noise due to logarithmic measurements were excluded in counts. Laboratory personnel who performed the blood assays were unaware of any subject’s clinical or laboratory data. Flow cytometry was performed using a Becton Dickinson FACSCalibur set and Cell Quest Pro software to detect fluorescence, forward scatter, and side scatter.

**Supporting results**

**S1 Fig. Human platelet-derived microparticles (PMPs)**


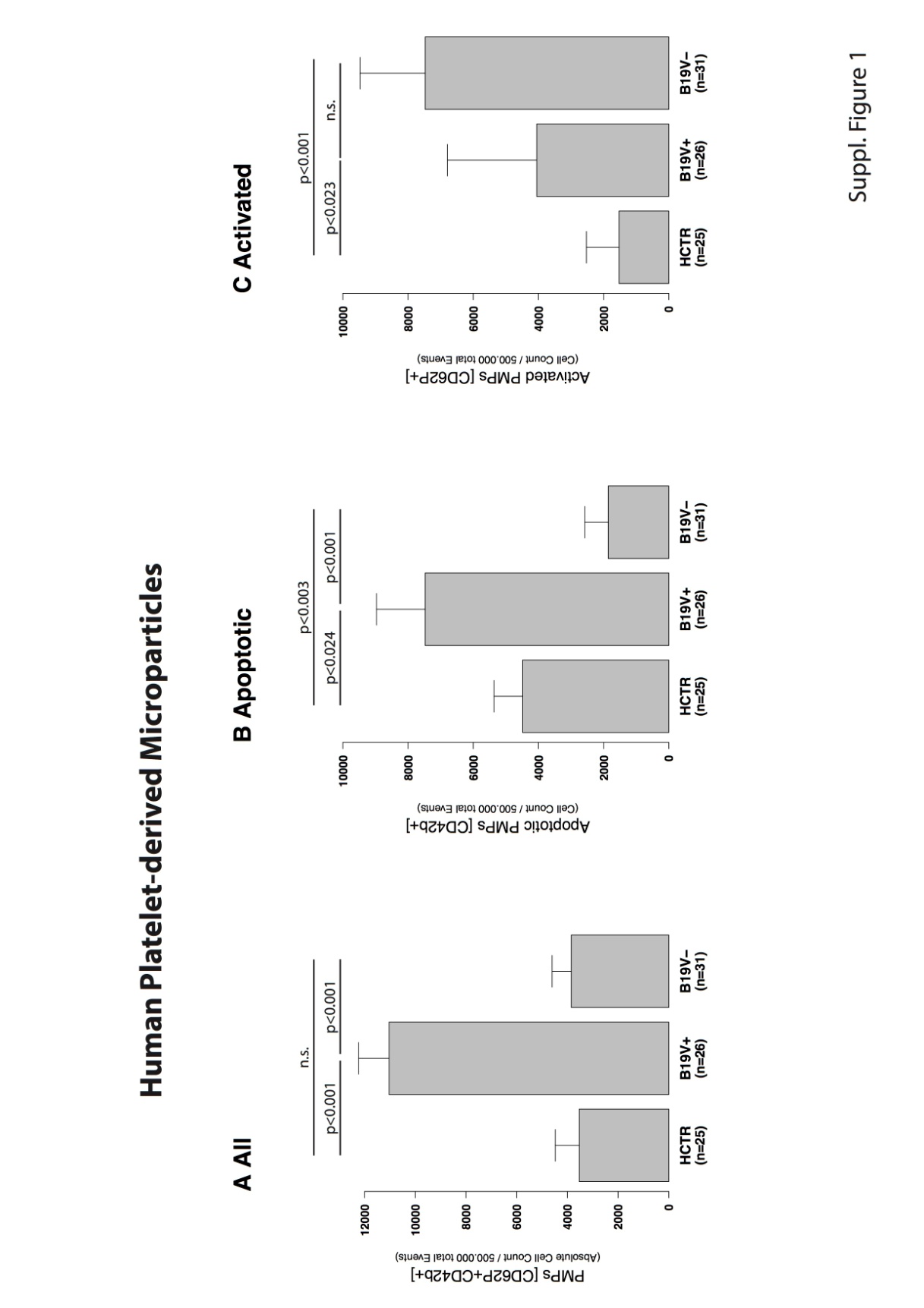


Human platelet-derived microparticles (PMPs) in patients with myocarditis divided into B19+ and B19V- patients and compared with age-matched healthy controls (HCTR). The B19V- group consisted of either no virus detection or HHV6+ and EBV+ samples. **A:** PMPs were significantly increased in B19V + patient samples compared to B19V- and HCTR. B19V- had increased EMP levels as well, but not significant versus HCTR. **B:** CD42b-AV+ PMPs represent apoptotic PMPs. Apoptotic PMPs were significantly higher detectable than activated PMPs in B19V+ and B19V-. **C:** CD62P+ PMPs represent activated PMPs.

**S2 Fig. Circulating inflammatory MPs.**


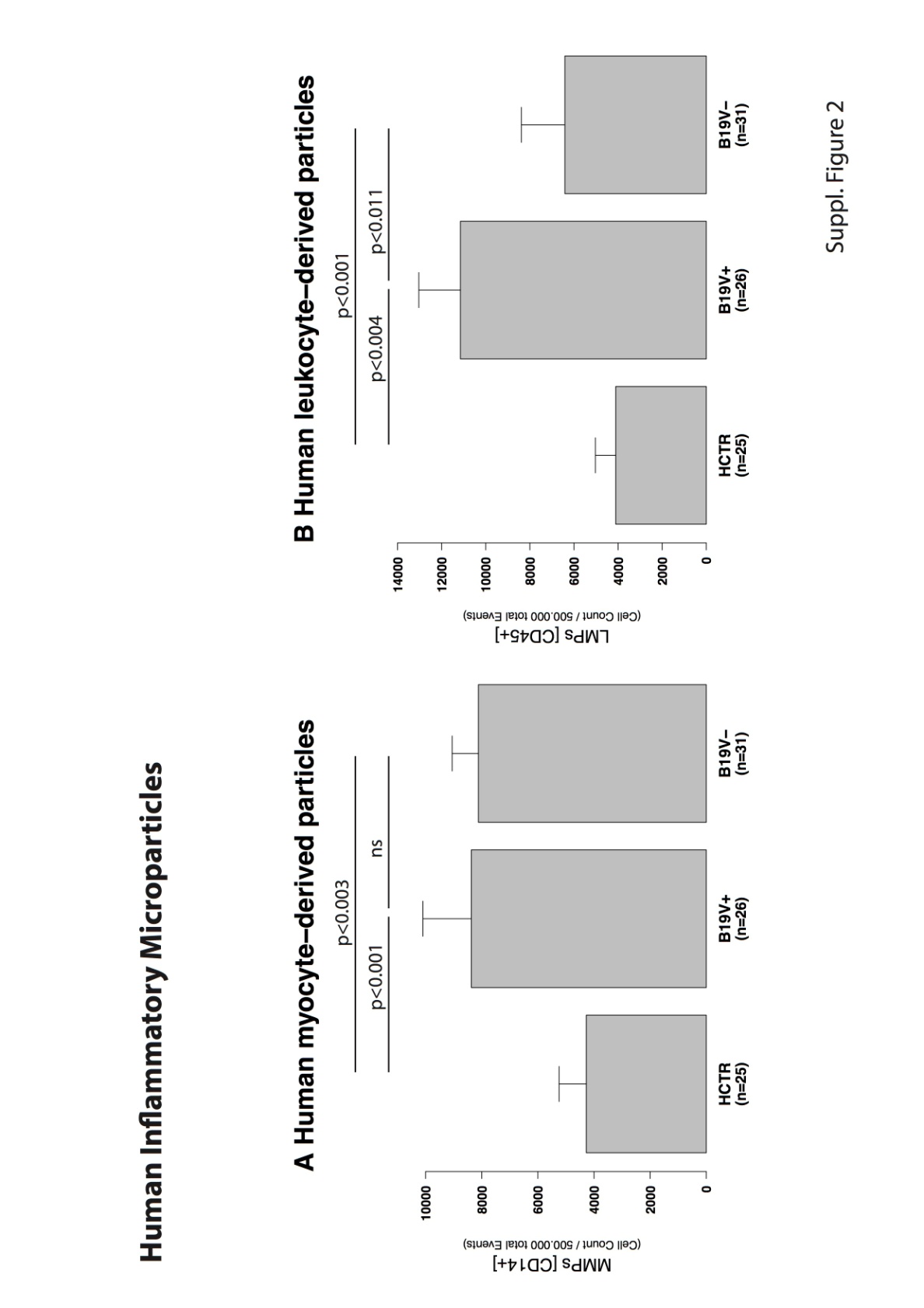


Monocyte-derived microparticles (MMPs,A) and leukocyte-derived microparticles (LMPs,B) in patients with myocarditis divided into B19V+ and B19V- patients and then compared with age-matched healthy controls (HCTR). The B19V- group consisted of either no virus detection or HHV6+ and EBV+ samples. **A:** MMPs were increased in both, B19V+ and B19V - in contrast to healthy controls (p<0.001 and p<0.003) but no significance between themselves. **B:** LMPs were significantly increased in B19V+ compared to B19V- (p<0.011) and healthy controls (p<0.004).
