# Supplementary figures and images for: Parvovirus B19-induced vascular damage in the heart is associated with elevated circulating endothelial microparticles
Source: PLoS One. 2017 May 22;12(5):e0176311. doi: 10.1371/journal.pone.0176311 (PMC5439674; doi:10.1371/journal.pone.0176311)

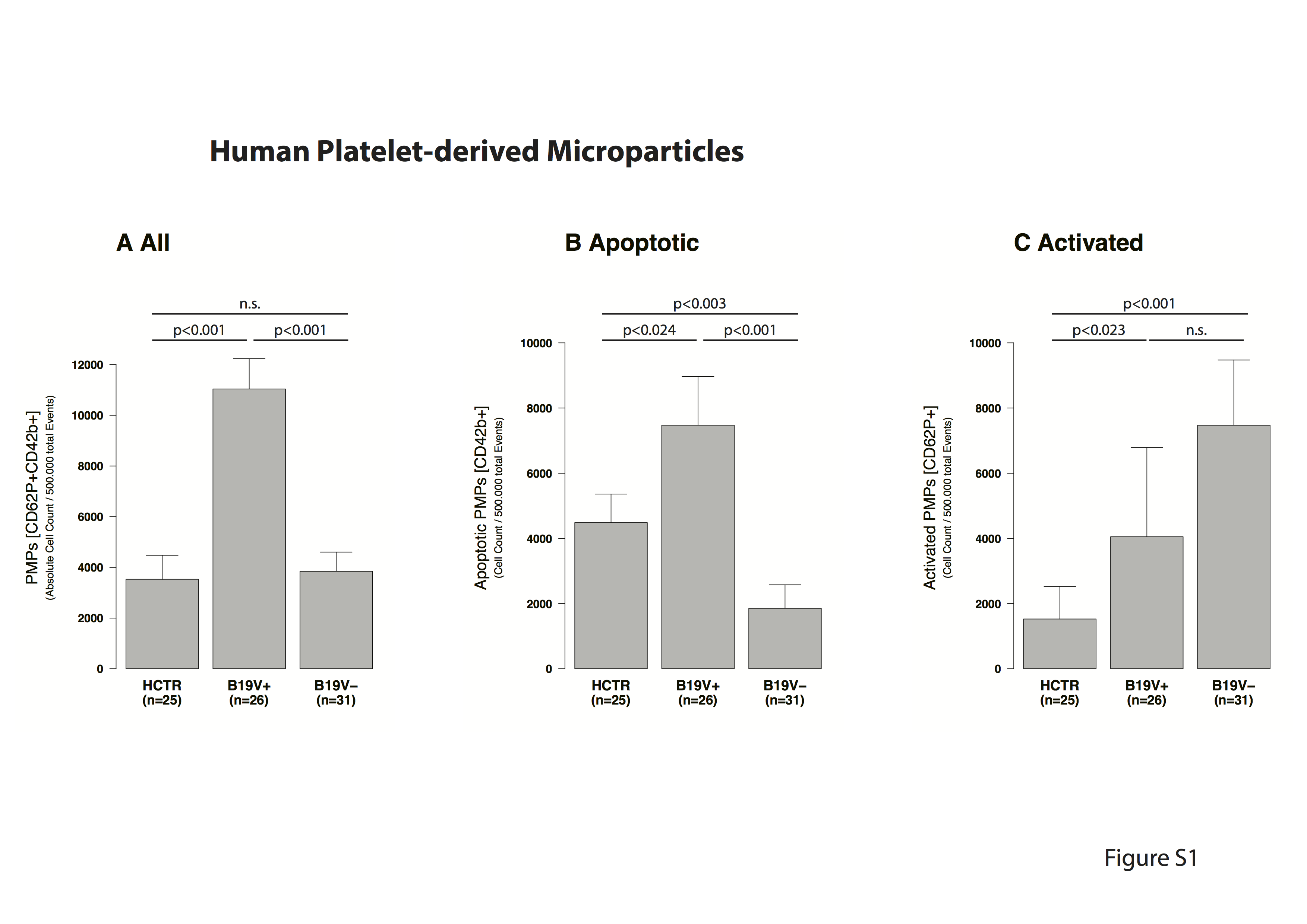

Supplement: S1 Fig — Human platelet-derived microparticles (PMPs) in patients with myocarditis divided into B19+ and B19V- patients and compared with age-matched healthy controls (HCTR). The B19V- group consisted of either no virus detection or HHV6+ and EBV+ samples. A: PMPs were significantly increased in B19V + patient samples compared to B19V- and HCTR. B19V- had increased EMP levels as well, but not significant versus HCTR. B: CD42b-AV+ PMPs represent apoptotic PMPs. Apoptotic PMPs were significantly higher detectable than activated PMPs in B19V+ and B19V-. C: CD62P+ PMPs represent activated PMPs. (TIFF) [file pone.0176311.s002.tiff]

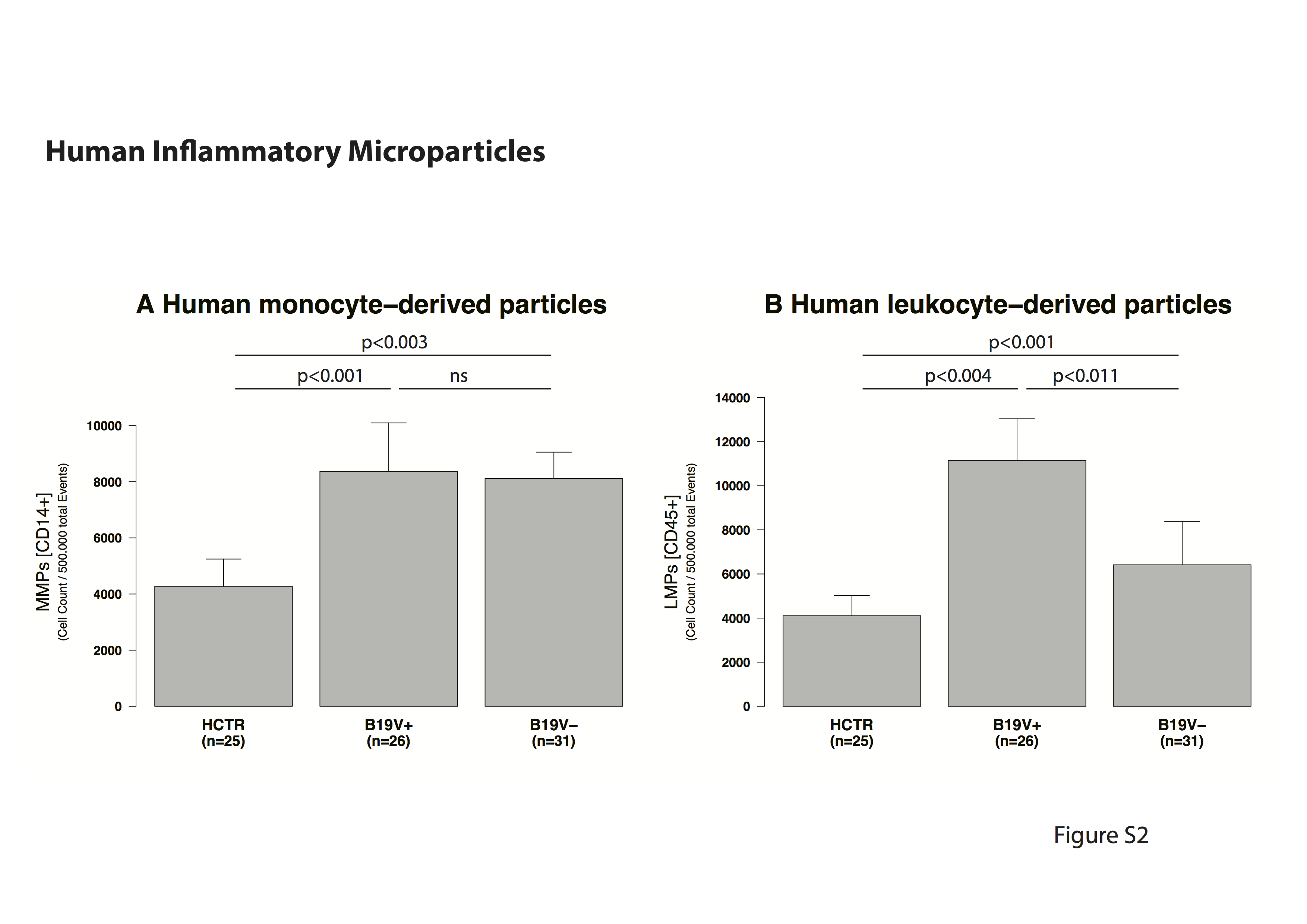

Supplement: S2 Fig — Monocyte-derived microparticles (MMPs,A) and leukocyte-derived microparticles (LMPs,B) in patients with myocarditis divided into B19V+ and B19V- patients and then compared with age-matched healthy controls (HCTR). The B19V- group consisted of either no virus detection or HHV6+ and EBV+ samples. A: MMPs were increased in both, B19V+ and B19V - in contrast to healthy controls (p<0.001 and p<0.003) but no significance between themselves. B: LMPs were significantly increased in B19V+ compared to B19V- (p<0.011) and healthy controls (p<0.004). (TIFF) [file pone.0176311.s003.tiff]
